# Small RNA molecules and their role in plant disease

**DOI:** 10.1007/s10658-018-01614-w

**Published:** 2018-10-29

**Authors:** Laura E. Rose, Elysa J. R. Overdijk, Mireille van Damme

**Affiliations:** 1 Institut für Populationsgenetik, Heinrich-Heine-Universität, Universitätsstraße 1, 40225 Düsseldorf, Germany; 2 Laboratory of Phytopathology, Wageningen University, P.O. Box 16, Wageningen, 6700 AA The Netherlands; 3 Laboratory of Cell Biology, Wageningen University, P.O. Box 633, Wageningen, 6700 AP The Netherlands; 4 Keygene N.V, Agro Business Park 90, 6708 PW Wageningen, The Netherlands

**Keywords:** Epigenetics, Plant immunity, Plant-pathogen interaction, RNA silencing

## Abstract

All plant species are subject to disease. Plant diseases are caused by parasites, e.g. viruses, bacteria, oomycetes, parasitic plants, fungi, or nematodes. In all organisms, gene expression is tightly regulated and underpins essential functions and physiology. The coordination and regulation of both host and pathogen gene expression is essential for pathogens to infect and cause disease. One mode of gene regulation is RNA silencing. This biological process is widespread in the natural world, present in plants, animals and several pathogens. In RNA silencing, small (20–40 nucleotides) non-coding RNAs (small-RNAs, sRNAs) accumulate and regulate gene expression transcriptionally or post-transcriptionally in a sequence-specific manner. Regulation of sRNA molecules provides a fast mode to alter gene activity of multiple gene transcripts. RNA silencing is an ancient mechanism that protects the most sensitive part of an organism: its genetic code. sRNA molecules emerged as regulators of plant development, growth and plant immunity. sRNA based RNA silencing functions both within and between organisms. Here we present the described sRNAs from plants and pathogens and discuss how they regulate host immunity and pathogen virulence. We speculate on how sRNA molecules can be exploited to develop disease resistant plants. Finally, the activity of sRNA molecules can be prevented by proteins that suppress RNA silencing. This counter silencing response completes the dialog between plants and pathogens controlling plant disease or resistance outcome on the RNA (controlling gene expression) and protein level.

## Introduction

‘A phytopathological study, [conveying] knowledge of a disease and the way to fight it, must be based on an understanding of the physiology of both the host plant and the parasite’ (Westerdijk 1917). Physiology includes the study of all the internal activities of an organism, including chemical, mechanical, and physical processes and the continuous communication between cells that occurs in the living organism and are required for the organism’s vital functions. Communication within and between cells within an organism is key, but during disease there is also communication between plants and pathogens and to prevent or limit disease, plants have developed a sophisticated immune system. Like Johanna’s successor stated: “Disease” is not a “state”, but a chain of processes involved in the changing interaction between the plant and its enemy; in other words, “disease” is a dynamic event (Kerling 1953).

How do plants and pathogens communicate? Plants and pathogens can communicate through an array of signals and molecules, including hormones, volatiles, proteins and nucleotides (including small noncoding RNAs, sRNAs). Most studied molecules that plants can recognize are peptides and proteins such as extracellular pathogen-associated molecular patterns (PAMPs) or intracellular pathogen effectors delivered into the host cells. Recognition by the plant triggers signal transduction events and this can lead to rapid defense responses that include massive transcriptional reprogramming within the plant. sRNA molecules, usually non-coding RNA molecules that are shorter than 200 nucleotide in length, can also lead to transcriptional reprogramming. sRNAs can form double-stranded RNA (dsRNA) that trigger post-transcriptional RNA silencing (PTGS) and lead to a decrease in messenger RNA level. RNA silencing is an epigenetic mechanism that initiates and sustains epigenetic changes, as do DNA methylation and histone modification. Epigenetic changes are heritable changes in gene expression that do not involve changes to the underlying DNA sequence; in other words: a change in phenotype without a change in genotype. Consequently epigenetic mechanisms provide a rapid mode to alter gene activity, e.g. when plants are exposed to parasites (Baulcombe 2004).

Genetic screens, particularly in Arabidopsis, have identified over 130 epigenetic regulating genes (reviewed by Pikaard and Mittelsten Scheid 2014). In Arabidopsis, at least 50 epigenetic regulators important for sRNA biogenesis and sRNA silencing have been identified, including DICER(DCL) and Argonaut (reviewed by Pikaard and Mittelsten Scheid 2014). In a nutshell, most sRNAs are generated by DCL proteins, the sRNAs are recruited by AGO proteins to form and function in an RNA-induced silencing complex (RISC).

In Arabidopsis four distinct RNA silencing pathways based on different types of sRNAs that originated from the activity of four dedicated DCL proteins were documented. DCL1 activity is important for microRNA (miRNA), DCL2 and DCL4 activity for natural antisense short interfering RNA (natsiRNA), DCL3 activity for repeat associated small interfering RNA (rasiRNA) and DCL4 activity for trans-acting small interfering RNA (tasiRNA) production (Eamens et al. 2008).

**Fig. 1:**
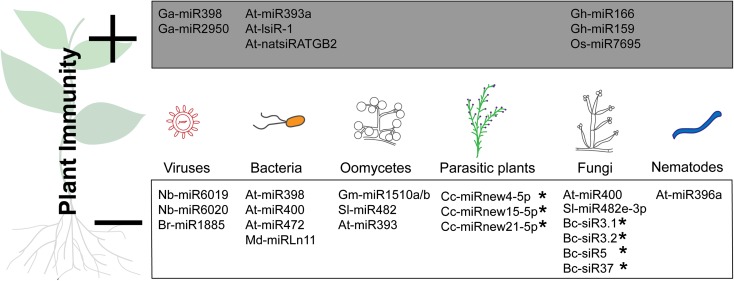
Identified small RNAs (RNA is abbreviated as R) that alter plant immunity. Six different parasites are depicted: viruses, bacteria, oomycetes, parasitic plants, fungi and nematodes. During the interaction with their host plants, various sRNAs were detected and shown to alter plant immunity. Most identified sRNAs originate from the plant, but a few sRNAs from parasites were also shown to alter plant immunity (indicated by *). sRNAs that enhance plant immunity are specified in the grey box by **+** and sRNAs that decrease plant immunity are specified by **-**. Abbreviations for sRNA according to species origin are: *Arabidopsis thaliana* (At), *Botrytis cinerea* (Bc), *Brassica rapa* (Br), *Cuscuta campestris* (Cc), *Gossypium arboreum* (Ga), *Gossypium hirsutum* (Gh), *Glycine max* (Gm), *Malus domestica* (Md), *Nicotiana benthamiana* (Nb), *Oryza sativa* (Os), *Solanum tuberosum* (St)

miRNAs are derived and excised from primary non-protein-coding *MIR* transcripts that form stem-loop structures (Meyers et al. 2008). In contrast to miRNAs, endogenous siRNAs are cleaved from long perfect dsRNAs, which are themselves products of specific RNA-dependent RNA polymerase (RDR) activities. Six RDRs are well studied in Arabidopsis (Wassenegger and Krczal 2006). RDRs were initially identified and studied due to their role in antiviral defence and RNA silencing (Schwach et al. 2005; Willmann et al. 2011). During viral infection, plants accumulate high levels of sRNAs (Baulcombe 2004). Yet RNA silencing factors are also important in plant responses to other pathogens. For example, Arabidopsis plants that lack RDR6 (*sgs2* or *rdr6* mutants) are more susceptible to the bacterium, *P. syringae* pv. *tomato* (*avrRpt2*), the fungus, *Verticillium dahliae*, and viruses, indicating that the production of sRNAs is important for immunity against multiple (or possibly all) pathogens (Mourrian et al.*,*^2000^, Katiyar-Agarwal et al. 2006, Ellendorff et al. 2009). Even more intriguing is the fact that the accumulated pathogenic sRNAs can indirectly influence the transcript levels of several RNA silencing factors such as AGO1, DCL1 and, DMT2. AGO1 transcripts are targeted and reduced by miRNA168. However the viral induced and accumulating sRNAs can outcompete miRNA168-AGO1 binding, resulting in reduced miRNA168-targeted degradation and accumulation of AGO1 transcript (Varallyay et al. 2010). DCL1 transcript is targeted by miRNA162. miRNA162 levels accumulate upon viral infection, and reduced levels of DCL1 transcript enhances viral susceptibility (Xie et al. 2003; Li et al. 2010). The DNA methyltransferase 2 (DMT2) transcript is targeted by miRNA773. miRNA773 levels accumulate upon bacterial infection, and reduced levels of DMT2 inhibit tumor formation during *Agrobacterium* infection (Crane and Gelvin 2007). Although miRNA168, miRNA162 and miRNA773 affect plant immunity, these miRNA are not included in Fig. 1, because these miRNA affect key factors of the RNA silencing machinery and, as such, will affect multiple cellular processes. For example, mutations in *Ago1* in *A. thaliana* can cause mild to severe morphological phenotypes, e.g. from aberrant leaves, to dwarfing, to nearly lethal. Deletion of DCL1 is less severe, probably because other DCL proteins can take over DCL1’s function, but nevertheless most *dcl1* mutants are affected in leaf morphology. DNA methylation was found to be necessary for proper embryo development and viability in Arabidopsis (Xiao et al. 2006). Because deregulation of RNA silencing factors cause severe morphological changes, the impact on plant immunity is likely to be a secondary effect. In this review, sRNAs (plant or pathogen derived) described thus far that regulate plant immunity by degrading transcripts that directly affect disease resistance are discussed. However, many of these sRNAs are also known to regulate other cellular processes, such as plant development and growth. The trade-off between regulating immunity, on the one hand, and cellular processes on the other is evident, but we focus on which and how sRNAs contribute to immunity. Furthermore, we discuss how sRNAs and their corresponding targeted gene transcripts that contribute to the plant-parasite dialog can be used to generate disease resistant plants.

### Known small RNAs can be exploited for disease resistance

Plants have an immune system to detect, respond to, and ward off disease caused by pathogens. sRNA molecules can originate from the host plant or from the parasite and can silence genes from the plant or parasite. In this review we discuss various types of sRNAs that alter plant immunity against six different types of parasites (Fig. 1). Depending on which genes are targeted and silenced, the sRNA can increase (indicated as **+** in Fig. 1) or reduce (indicted as **-** in Fig. 1) plant immunity. Most of the sRNAs in Fig. 1 originate from the host plant and target gene transcripts within the host plant. sRNAs indicated with asterisks originate from the parasite and target gene transcripts within the host plant. The details of the various sRNAs, e.g. origin of sRNA, involved parasites, origin and name of target transcripts, effect on immunity, and references are specified in Table 1. When accumulation of a sRNA results in a positive effect on plant immunity, causing enhanced resistance, the sRNAs are positioned in the grey box (Fig. 1) and in grey shaded rows (Table 1). sRNA accumulation that results in a negative effect on plant immunity is indicated by the open box (Fig. 1) and rows (Table 1) without shading. Accumulation of a single plant sRNA can cause reduced resistance to multiple parasites, e.g. At-miR400 accumulation leads to reduced resistance against the bacteria *Pseudomonas syringae* pv. *tomato* DC3000 and the fungus *Botrytis cinerea* in *A. thaliana* (Park et al. 2014, Fig. 1). The targeted transcripts of the sRNAs that reduce resistance are often of genes known to be important for resistance. Resistance genes (R-genes) are targeted by eight (in bold) of the 26 sRNAs presented in Table 1. If the origin of the sRNA and the regulated gene transcript is different, the sRNA is hypothesized to be transferred between organisms, also termed inter-kingdom translocation (Table 1, marked as yes in seventh column). Plant sRNAs that target viral gene transcripts do not translocate between both organisms, because the targeting of the viral gene transcripts takes place inside the plant cell (Table 1, marked as a dash in seventh column). Accumulation of eight sRNAs has a positive effect on immunity causing enhanced disease resistance (Fig. 1 and Table 1, shaded in grey). These sRNAs silence genes that encode for auxin receptor transcripts, negative defence regulators, or putative susceptibility factors from host plants or factors from the parasite that are relevant for a parasite to cause disease (Table 1).

**Table 1:** Identified sRNAs and targeted gene transcripts of various plant diseases. Type of sRNA (RNA is abbreviated as R) including two letter abbreviation of the species of origin (first column). Other columns indicate: origin of sRNA, involved parasites, name and origin of sRNA targeted gene transcript, effect on plant immunity by sRNA, if inter-kingdom translocation occurs and references

sRNA	sRNA origin	Involved pathogen	Target transcript	Target origin	Effect of sRNA on immunity	Inter-kingdom transloca-tion	Reference
At-miR393a	*Arabidopsis thaliana*	Bacteria, *Pseudomonas syringae* pv. tomato DC3000 and oomycete, *Hyaloperonospora arabidopsidis*	auxin receptor transcripts (At-*TIR1*, At-*AFB2*, At-*AFB3*)	Plant, *Arabidopsis thaliana*	positive	no	Navarro et al. 2006 Robert-Seilaniantz et al. 2011
At-miR396a/b	*Arabidopsis thaliana*	Nematodes, *Heterodera schachtii*	growth-regulating factor (At-*GRF1* and At-*GRF3*)	Plant, *Arabidopsis thaliana*	negative	no	Hewezi et al. 2012
At-miR398	*Arabidopsis thaliana*	Bacteria, *Psuedomonas syringae*	two copper superoxide dismutases (At-*CSD1,CSD2*) and a cytochrome c oxidase subunit V (At-*COX5*)	Plant, *Arabidopsis thaliana*	negative	no	Li et al. 2010 Jagadeeswaran et al. 2009
At-miR400	*Arabidopsis thaliana*	Bacteria, *Psuedomonas syringae* DC3000 and fungus, *Botrytis cinerea*	peptatricopeptide repeat (At- *PPR1* and *PPR2*)	Plant, *Arabidopsis thaliana*	negative	no	Park et al. 2014
**At-miR472**	*Arabidopsis thaliana*	Bacteria, *Pseudomonas syringae* pv. *tomato* DC3000	TIR-NB_LRR and CC-NB-LRR resistance genes, including At-*RPS5*	Plant, *Arabidopsis thaliana*	negative	no	Boccara et al. 2014
At-lsiR-1	*Arabidopsis thaliana*	Bacteria, *Pseudomonas syringae* (*avrRpt2*)	RAP-domain protein involved in disease resistance (*AtRAP*)	Plant, *Arabidopsis thaliana*	positive	no	Katiyar-Agarwal et al. 2007
At-natsiR ATGB2	*Arabidopsis thaliana*	Bacteria, *Pseudomonas syringae* (*avrRpt2*)	pentatricopeptide repeat protein-like gene, a putative negative regulator of resistance gene RPS2 At-*PPRL*	Plant, *Arabidopsis thaliana*	positive	no	Katiyar-Agarwal et al. 2006
**Br-miR1885**	*Brassica rapa*	Virus, Turnip mosaic virus (TuMV)	TIR-NB-LRR, resistance genes	Plant, *Arabidopsis thaliana* and *Brassica rapa*	negative	no	He et al. 2008
Bc-siR3.1, siR5	*Botrytis cinerea*	Fungus, *Botrytis cinerea*	At-*MPK2,* At-*MPK1,* At-*PRXIIF,* Sl-*MAPKKK4*	Plant, *Arabidopsis thaliana* and *Solanum lycopersicum*	negative	yes	Weiberg et al. 2013
Bc-siR3.2	*Botrytis cinerea*	Fungus, *Botrytis cinerea*	At-*MPK2,* At-*MPK1,* At-*PRXIIF,* Sl-*MAPKKK4*	Plant, *Arabidopsis thaliana* and *Solanum lycopersicum*	negative	yes	Weiberg et al. 2013
Bc-siR5	*Botrytis cinerea*	Fungus, *Botrytis cinerea*	At-*MPK2,* At-*MPK1,* At-*PRXIIF,* Sl-*MAPKKK4*	Plant, *Arabidopsis thaliana* and *Solanum lycopersicum*	negative	yes	Weiberg et al. 2013
Bc-siR37	*Botrytis cinerea*	Fungus, *Botrytis cinerea*	At-*WRKY7*, At-*PMR6*, At-*FEI2*	Plant, *Arabidopsis thaliana*	negative	yes	Wang et al. 2017
Cc-miR new4-5p	*Cuscuta campestris*	Parasitic plant, *Cuscuta campestris*	new4:At-*SEOR1*	Plant, *Arabidopsis thaliana*	negative	yes (?)	Shahid et al. 2017
Cc-miR new15-5p	*Cuscuta campestris*	Parasitic plant, *Cuscuta campestris*	new15:At-*BIK1*	Plant, *Arabidopsis thaliana*	negative	yes (?)	Shahid et al. 2017
Cc-miR new21-5p	*Cuscuta campestris*	Parasitic plant, *Cuscuta campestris*	new21: At-*TIR1/AFB2/AFB3*	Plant, *Arabidopsis thaliana*	negative	yes (?)	Shahid et al. 2017
Ga-miR398	*Gossypium arboreum*	Virus, *Cotton leaf curl Multan virus*	multiple Open Reading Frames	Virus	positive	–	Akmal et al. 2017
Ga-miR2950	*Gossypium arboreum*	Virus, *Cotton leaf curl Multan virus*	multiple Open Reading Frames	Virus	positive	–	Akmal et al. 2017
Gh-miR159	*Gossypium hirsutum*	Fungus, *Verticillium dahliae*	isotrichodermin C-15 hydroxylase (Vd-*HiC15*)	Fungus, *Verticillium dahliae*	positive	yes	Zhanga and Zhao 2016
Gh-miR166	*Gossypium hirsutum*	Fungus, *Verticillium dahliae*	Ca^2+^-dependent cysteine protease (Vd-*Clp1*)	Fungus, *Verticillium dahliae*	positive	yes	Zhanga and Zhao 2016
**Gm-miR1510a/b**	*Glycine max*	**Oomycete,** ***Phytophthora sojae***	TIR-NB-LRR resistance genes	Plant*, Glycine max*	negative	no	Cui et al. 2017
**Md-miRLn11**	*Malus domestica*	Bacteria, *Alternaria alternata f.sp.mali*	NB-LRR resistance gene	Plant, *Malus domestica*	negative	no	Ma et al. 2014
**Nb-miR6019**	*Nicotiana benthamiana*	Virus, tobacco mosaic virus (TMV)	TIR-NB-LRR immune receptor *N*	Plant, *Nicotiana benthamiana*	negative	no	Li et al. 2012
**Nb-miR6020**	*Nicotiana benthamiana*	Virus, tobacco mosaic virus (TMV)	TIR-NB-LRR immune receptor *N*	Plant, *Nicotiana benthamiana*	negative	no	Li et al. 2012
Os-miR7695	*Oryza sativa*	Fungus, *Magnaporthe oryzae*	member of the metal ion transporter family, Os-*Nramp6*	Plant, *Oryza sativa*	positive	no	Campo et al. 2013
**Sl-miR482a /f /f’**	*Solanum lycopersicum*	Oomycete, *Phytophthora infestans*	*NBS-LRR* like gene	Plant, *Solanum lycopersicum*	negative	no	de Vries et al. 2018
**Sl-miR482 e-3p**	*Solanum lycopersicum*	Fungus, *Fusarium oxysporum f. sp. lycopersici*	*NBS-LRR* like gene, Sl*-FRG3*	Plant, *Solanum lycopersicum*	negative	no	Ji et al. 2018

The following abbreviations are used: Toll and interleukin-1 receptor domain- nucleotide binding domain-leucine-rich repeat protein (TIR-NB-LRR), coiled coil- nucleotide binding domain- leucine-rich repeat protein (CC-NB-LRR), mitogen-activated protein kinases (MAPK), mitogen-activated protein kinase kinase kinase (MAPKKK), transport inhibitor response (TIR), auxin binding protein (AFB), resistance against *Pseudomonas syringae* (RPS), peroxiredoxin-2F (PRXIIF), powdery mildew resistance (PMR), Leucine-rich repeat receptor-like kinases (FEI, named after the Chinese word corresponding to fat), Natural resistance-associated macrophage protein 6 (Nramp6, a member of the metal ion (Mn^2+^-iron) transporter family)

### Why exploit small RNA molecules for disease resistance?

In addition to the fact that several sRNAs contribute to immunity (Fig. 1 and Table 1), additional reasons to exploit sRNAs to generate disease resistant plants are: (1) sRNAs can move through and between organisms, (2) sRNAs act fast and can alter gene expression of multiple transcripts at once, (3) sRNAs and their targets are conserved between various organisms, and (4) prospects for application of sRNA based plant disease resistance are promising.

#### sRNA molecules can move

**Fig. 2:**
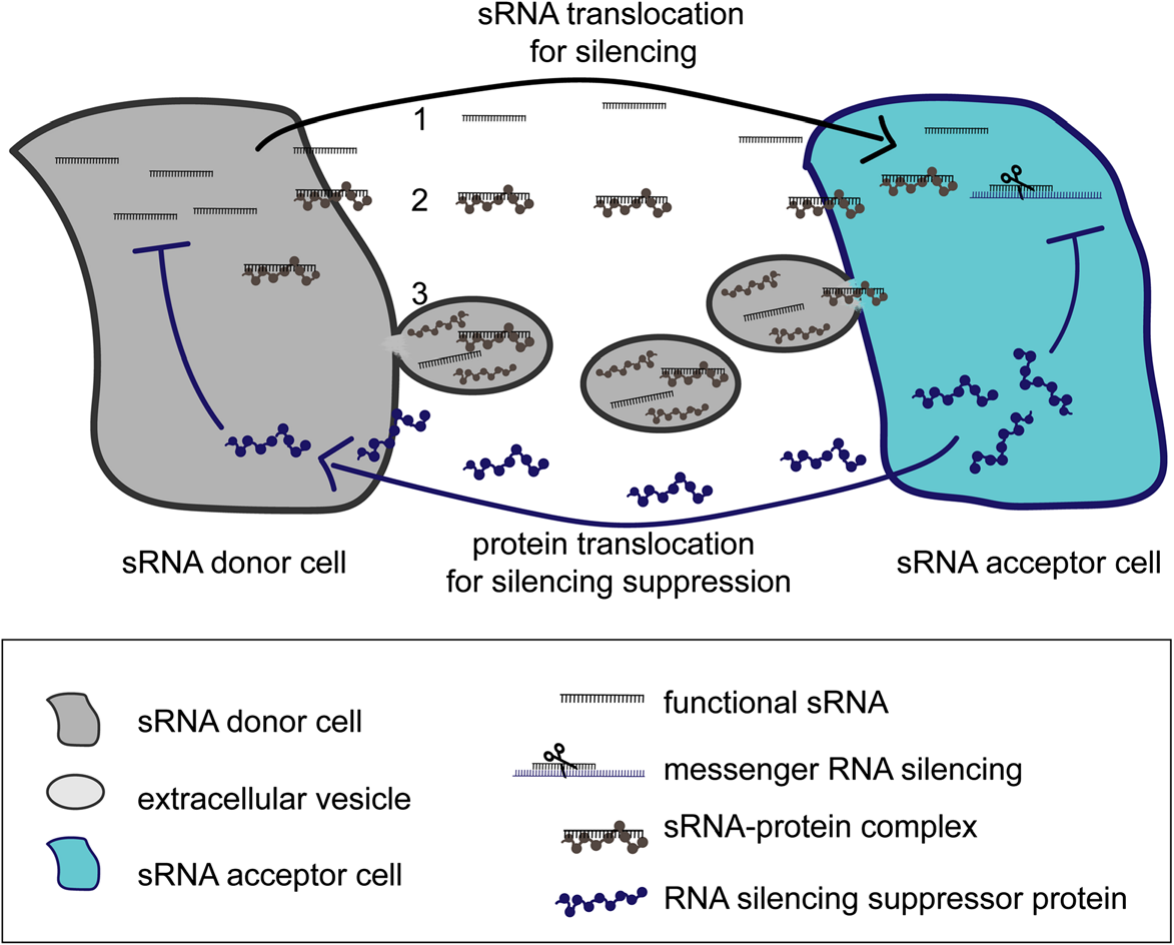
Potential routes for inter-kingdom RNA silencing. Three hypothetical scenarios (1–3) for sRNA translocation between cells of two different species are depicted. In grey, a sRNA donor cell and in blue, a sRNA acceptor cell, the direction of sRNA translocation is shown by the grey arrow. Three possible translocation scenarios are (1) sRNA on its own (naked), (2) sRNA as part of an RNA-protein complex (in grey) and (3) sRNA is loaded within an extracellular vesicle (EV). The blue arrow on the bottom depicts the translocation of RNA silencing suppressor proteins (in blue) from the sRNA acceptor cell in the direction of the sRNA donor cell. The RNA silencing suppressor protein prevents sRNA based messenger RNA silencing, e.g. by preventing sRNA accumulation in the donor and/or in the acceptor cell

RNA molecules, including sRNAs, have been found to be mobile within organisms, allowing gene silencing between cells and tissues. By grafting wild type shoots to *dcl2,3,4* mutant roots of Arabidopsis, it was shown that sRNAs that are produced in the shoot move towards the roots via the vascular system (Molnar et al. 2010). sRNAs can also be translocated between organisms and lead to gene silencing, termed trans-kingdom RNA silencing. Trans-kingdom RNA silencing was originally explored to generate disease resistance in wheat and barley against the powdery mildew fungus, *Blumeria graminis*. Wheat and barley plants that produced dsRNA or anti-sense RNA fragments that were designed to affect gene expression in the fungus were shown to silence the fungal genes (Nowara et al. 2010). This form of trans-kingdom RNA silencing is called host induced gene silencing (HIGS). Likewise, two plant miRNAs were shown to silence *V. dahliae* transcripts that are important for virulence (Zhang^a^ and Zhao 2016) (Fig. 1). Another example of naturally occurring trans-kingdom RNA silencing was shown by sRNAs that originated from the fungus *B.cinerea*, Bc-sR3.1, Bc-sR3.2, Bc-sR5 and Bc-sR37 (Fig. 1, Table 1, Wang et al. 2017, Weiberg et al. 2013). These fungal sRNAs are translocated to the plant and silence Arabidopsis and tomato genes involved in immunity (Weiberg et al. 2013, Fig. 1, Table 1). Also more recently, sRNAs from *V. dahliae* were shown to function in plants and silence genes involved in immunity (Wang et al. 2016). So sRNAs from fungi can target and silence plant transcripts, but also sRNAs from plants can target and silence pathogen transcripts. Finally, sRNAs from the parasitic plant *C. campestris* can translocate and target transcripts in *A. thaliana*. Although this is not a trans-kingdom RNA silencing act, this cis-kingdom translocation illustrates how powerful the sequence specific regulation of transcripts by sRNAs is. Because sRNA silencing relies on nucleotide matching, and a minor difference between the sRNA target site in the donor versus the acceptor plant target sequence is sufficient to discriminate between ‘self’ and ‘foreign’ (Shahid et al. 2017). While the evidence for trans-kingdom RNA silencing continues to accumulate, it is still unknown how sRNAs are translocated between organisms, e.g. the export from the sRNA producing cell and the import into the sRNA acceptor cell. Presumably sRNAs that move between organisms could rely on mechanisms similar to those observed for extracellular transport within an organism. Figure 2 depicts a model for three possible sRNA translocation scenarios: (1) sRNA on its own (naked), (2) sRNA as part of a RNA-protein complex, and (3) sRNA within an extracellular vesicle (EV). However, the first scenario, translocation of naked RNA is expected to work only between cells of a single organism, e.g. sRNAs translocation via a gap-junction (animal cells) or plasmodesmata (plant cells). The movement of naked sRNAs between cells from different organisms is unlikely due to the presence of ribonucleases (RNases, including exoribonuclease enzymes that degrade miRNA) in the extracellular space (Ramachandran and Chen 2008). The second scenario posits the translocation of sRNAs as part of a RNA-protein complex, independent of vesicles. In fact, miRNA-AGO2 complexes were abundantly present and strongly nuclease- and protease-resistant in human blood plasma and cell cultures (Arroyo et al. 2011; Turchinovich et al. 2011). Therefore, translocation of sRNAs as part of a RNA-protein complex may also occur between plants and fungi.

Evidence for the third scenario, sRNA translocation by EVs, is accumulating. Eukaryotic cells secrete two main classes of EVs: microvesicles and exosomes. Microvesicles are 100–1000 nm in diameter and exosomes are smaller, 30–150 nm in diameter. Exosomes originate from multivesicular bodies that fuse with the plasma membrane and microvesicles are formed by direct shedding from the plasma membrane (Gyorgy et al. 2011). However, since most studies have not clearly defined the origin of EVs under study, we will refer to EVs rather than microvesicles or exosomes. Most studies so far have focussed on mammalian EVs and less is known about the production, regulation and function of plant EVs. However, the presence of EVs during plant infection in the extrahaustorial matrix and in the papillary matrix was shown by electron microscopy (Micali et al. 2011; Politis and Goodman 1978). Recently, plant EVs were isolated from Arabidopsis and sunflower leaves by differential centrifugation (Regente et al. 2017; Rutter and Innes 2017). The size of these vesicles ranged between 50 and 300 nm and the presence of putative exosome marker PEN1 indicates that exosomes are part of this plant EV population (Rutter and Innes 2017). The fact that the secretion of EVs is enhanced upon infection with the bacterial pathogen *Pseudomonas syringae* and that the EV proteome is enriched for defence-related proteins, bolsters the importance of EVs during plant immunity (Regente et al. 2017, Rutter and Innes 2017). sRNA sequencing from human, mammalian, insect and fungal EVs has led to the identification of sRNAs and components of the silencing machinery inside EVs, which makes it likely that plant EVs also contain sRNAs that could contribute to trans-kingdom RNA silencing between plants and fungi (Lefebvre 2017; Han and Luan 2015). And indeed, during the revision of this manuscript, Cai and colleagues (Cai et al. 2018) reported that *A. thaliana* cells secrete exosome-like EV to deliver sRNAs into the fungal pathogen *B. cinerea* and that the delivered *A. thaliana* sRNAs silence fungal virulence transcripts (Cai et al. 2018).

However many questions remain open. For example, what determines the direction and specificity during EV exchange (Boevink 2017)? How is the cargo, in this case specific sRNAs, directed to EVs from within the donor cell? Furthermore, it is plausible that the composition of the EV cargo will depend on the cell type (e.g. origin) and cell status (e.g. development or stress), adding an additional layer of complexity. In humans, there is some evidence for the selective loading of miRNAs into vesicles, which results in the transfer of miRNAs from macrophages to acceptor endothelial cells (Squadrito et al. 2014). Nevertheless, the loading of cargo into the EVs is probably passive and based on endogenous levels of natural targets, because high levels of natural targets limit miRNA levels as cargo of the secreted EVs (Squadrito et al. 2014). An active selection mechanism has been proposed through the unidirectional transfer of miRNAs between T-cells and antigen-presenting cells during antigen recognition of the human immune response (Mittelbrunn et al. 2011).

Additional questions are: Why do some EVs burst in the apoplastic space and are others taken up by the acceptor cell? How are EVs taken up by the acceptor cell? Recent evidence suggest that EVs are endocytosed by the acceptor cell: sunflower EVs labelled with the membrane dye FM4–64 were mixed with fungal spores and this dye ended up inside the fungal spores (Regente et al. 2017). Although the exact mode of sRNA translocation is still under investigation, the movement of sRNAs is evident and required for the spreading of RNA silencing and, as such, controlling gene regulation within and between organisms.

#### Cluster bomb efficiency: sRNAs act fast and can alter gene expression of multiple transcripts at once

Plants are sessile organisms and rely on a fast response to combat disease caused by pathogens. Via the RNA silencing mechanism, the abundance of gene transcripts can be altered quickly resulting in a change in gene activity, independent of genomic mutations. Most sRNAs in Fig. 1 alter gene transcript levels by degrading the messenger RNA (mRNA). One exception is AtlsiR-1, which is predicted to destabilize targeted mRNA through decapping, leading to XRN4-mediated 5′-to-3′ degradation (Katiyar-Agarwal et al. 2007). XRN4 is a cytoplasmic exoribonuclease that participates in the degradation of mRNAs (Souret et al. 2004). So sRNAs can alter gene expression quickly and efficiently, allowing for a swift response to the invading parasites. In addition, a single sRNA can alter multiple transcripts at once. The regulation of these specific transcripts can be important for different cellular processes, but several sRNAs are known to reduce the levels of multiple R-genes at once. One example is miR482/2118 which can regulate the expression of a major class of R-genes, nucleotide-binding site leucine-rich repeats (NBS-LRRs) (Shivaprasad et al. 2012). The silencing of multiple transcripts can be attributed to the silencing by a single sRNA or by a cascade of silencing effects. The initiator of this silencing cascade can be a single miRNA which leads to the production of multiple secondary endogenous sRNAs, termed siRNAs, from one transcript, and these siRNAs in turn can target multiple transcripts. A well-studied class of these siRNAs are the tasiRNAs. tasiRNAs are generated from TAS gene-derived transcripts by miRNA based transcript degradation. *A. thaliana* has four families of TAS transcripts, of which TAS1 and TAS2 are targeted by At-miR173, TAS3 by At-miR390 and TAS4 At-miR828, leading to tasiRNA production (reviewed by Fei et al. 2013). Secondary sRNAs can also be produced from protein coding transcripts, not only from known protein coding transcripts like TAS genes. To summarize, a single sRNA can shut down multiple transcripts with a similar target sequence embedded in their genetic code simultaneously, and through the production of phasi and tasiRNA, a single sRNA can produce a wide diversity of sRNAs that target and silence even more gene transcripts. Because sRNA-based gene silencing is fast, efficient and can regulate multiple target transcripts at once, it is essential to explore and clarify the role of sRNAs in immunity.

#### Co-evolution between sRNAs and target transcripts: sRNAs and their targets are conserved between various organisms

Efficient RNA silencing depends on the similarity between the sRNA sequence and the target site sequence of the regulated gene. However, due to the natural and unavoidable introduction and occasional fixation of nucleotide mutations in natural populations, neither the miRNA gene, nor its targets, are immune to evolutionary change. This means that the sequence evolution of miRNAs is constrained by the need to maintain specificity and consistency of targeting in the face of its own continual sequence evolution and that of their target genes. This sets up an intriguing co-evolutionary dynamic within the organism’s own genome, to maintain consistent negative regulation, while accommodating both adaptive and neutral evolutionary changes (for example at synonymous coding positions) of the target genes. This evolutionary dilemma applies for miRNAs that target developmental processes, as well as for miRNAs that target immune systems. However, if host fitness is dependent upon faithful targeting of immune system genes such as R-genes, this is no easy task. Unlike most genes controlling development, R-genes are notorious for their rapid, adaptive protein evolution and in this sense, act as a moving target for their corresponding miRNAs (Rose et al. 2004; Clark et al. 2007). For this reason miRNAs, whether specific for the immune system or a developmental pathway, typically converge on conserved regions of their target genes. One of the best studied examples of miRNAs that target R-genes is the miR482 family (Shivaprasad et al. 2012). The target site of the miR482 gene family resides in the P-loop, present within the NBS region of R-genes (Shivaprasad et al. 2012). This functionally conserved region evolves more slowly at the protein level than other regions of these R-genes, and thereby can serve as a consistent, reliable target site (Zhangb et al. 2016). Although the amino acid sequence of this region is conserved, synonymous substitutions still accumulate in this region over time. The accumulation of synonymous differences at the P-loop among closely related R-genes appears to have been matched by sequence divergence among members of the miR482 family (Shivaprasad et al. 2012; Zhangb et al. 2016). Variation in the mature miRNA sequence among members of the same miRNA family is typically concentrated in the sites corresponding to the third positions in the codons of the R-gene target (Shivaprasad et al. 2012; Zhangb et al. 2016). This ensures consistent targeting, despite sequence divergence at synonymous positions of the R-gene targets.

The miR482 gene family is present across land plants and therefore represents an ancient and conserved form of negative regulation of R-genes (Shivaprasad et al. 2012; de Vries et al. 2015; Zhangb et al. 2016; Ji et al. 2018). The size of the gene family varies across plant species, from 1 to 24 (de Vries et al. 2015). Within a given plant species, the proportion of targeted R-genes also varies. However, we observe that among the closely related species, such as tomato and potato, approximately 20% of the R-genes are predicted to be targeted by members of the miR482 family (de Vries et al. 2015). In some cases, closely related species encode the same mature miRNAs. In other cases, sequence divergence between species at orthologous *MIR* genes leads to different mature miRNAs. In this respect, the miR482 gene family appears to be fairly labile, showing a mixture of evolutionary rates and constraints across gene members (de Vries et al. 2015). To what degree these different evolutionary patterns reflect the evolutionary history of their targets has not been systematically evaluated (Zhang^b^ et al. 2016). On-going studies comparing the sequence evolution of the targets, the miRNAs and their targeting behavior will shed light on the factors underlying the dynamic evolutionary history of miRNAs.

Understanding the evolutionary history of miRNAs and their targets can be useful for assessing their potential for plant protection. For example, one consequence of the slow rate of amino acid evolution in particular regions of R-genes, such as the P-loop, is that the same target sequence is often found in R-genes within and between plant species. This means that miRNA-targeting can regulate multiple transcripts within a single individual and can be functional across species boundaries. This opens the possibility of modifying resistance responses across a wide taxonomic range through by the expression of a single molecule. Of course, this assumes that immunity can be enhanced in the presence of the miRNA, which may be the case if the target is a host susceptibility factor, a negative regulator of host defense or an effector transcript from the pathogen. However if the miRNA reduces immunity, the target site in the regulated R-gene could be altered to prevent R-gene silencing and enhance the resistance response. In either case, adequate genome information from the targeted individual is important to ascertain the extent of intended targeting and/or undesirable off-targeting. For many crop species and their pathogens, extensive genomic information is readily available or is being generated, so this becomes a more straightforward exercise. In summary, the discovery of this form of negative gene regulation which is intimately tied to plant disease resistance has given us an additional potential tool to fight pathogens.

#### Prospects to exploit sRNA based pathogen resistance

The advantage and feasibility for agronomists to temporarily shut off the activity of a gene by RNA silencing has already been successfully demonstrated. Spray application of dsRNA on potato plants matching beetle-specific gene sequences led to silencing of vital insect genes, resulting in disease resistant potato plants (Palli 2014, Miguel and Scott 2016). Currently, multiple companies invest in spray application of dsRNAs to cure plants of diseases caused by foliar pathogens (Miguel and Scott 2016, Regalado 2015). This novel plant protection method can potentially reduce our reliance on chemicals and environmentally harmful pesticides.

Another advantage of this method is that it is not considered to be a GMO (genetically modified organism) approach. sRNAs that arrest parasites and promote disease resistance could be applied directly to crops (Kamthan et al. 2015). The use of sRNAs is also more attractive because sRNAs are expected to have greater specificity and many naturally occur in plants. However, two major drawbacks for spray application of sRNA are: the fast degradation of applied (naked) sRNAs and the high cost to produce sufficient quantities of the sRNA molecules. A protected form of sRNA could help address the first challenge, e.g. sRNAs as a coating on clay particles or sRNAs loaded in a synthetic vesicle (Mitter et al. 2017; Schmitt et al. 2016). The application of dsRNA embedded to double hydroxide clay nanosheets (BioClay) afforded virus protection for at least 20 days on tobacco plants (Mitter et al. 2017). The cost to produce sRNAs is currently falling from over $100,000 per gram a few years ago to $2 per gram currently (Le Page 2017). Besides using vesicle or clay particle based delivery for foliar parasites, this method can also be used against root invading parasites, e.g. applied directly to the roots via watering or by seed coating. Furthermore, direct application of sRNA molecules to combat plant disease is substantially less time consuming and less complicated than generating genetically modified plants.

### sRNA targeted transcripts that alter plant immunity

sRNAs can alter plant immunity depending upon the action of their targets. Disease susceptibility can be enhanced following the accumulation of parasite or plant sRNAs that silence plant genes important for resistance (Table 1). Eight out of 28 sRNAs indicated in Table 1 target and silence classical R-genes. Additionally, several fungal and plant sRNAs target other types of plant genes that are also important for plant immunity, e.g. MAP kinases and WRKY transcription factors (Table 1). sRNAs may also enhance susceptibility by silencing avirulence genes of the parasite, but no examples of that have been reported thus far.

On the other hand, resistance can be enhanced when sRNA accumulate which silence plant “susceptibility” genes and/or genes that are required for pathogen virulence. Nine plant sRNAs that enhance disease resistance against pathogens and three sRNAs that enhance resistance against plant parasites have been reported (Table 1). Although the plant-targeted genes are not classical susceptibility genes, silencing or loss of these genes results in enhanced disease resistance. It would be interesting to verify if classical susceptibility genes, e.g. *DMR1* or *DMR6*, are also regulated by sRNAs during downy mildew infection (van Damme et al. 2008 and van Damme et al. 2009).

Silencing of viral gene transcripts by plant sRNAs proceeds inside the plant cell, however the silencing of three fungal genes by two plant miRNAs is suggested to take place in the fungus. Absence or reduction of these fungal genes affects the virulence and fitness of the fungus, *Verticillium dahliae* (Table 1).

Although sRNAs that enhance disease resistance can be used directly to generate disease resistant plants (shaded box Fig. 1) in contrast to sRNAs that deregulate immunity and lead to susceptibility (unshaded boxes of Fig. 1), both should be explored, because both impinge on plant immunity and are central to the regulation and communication between plant and pathogen. For example, sRNAs that downregulate R-genes or the target sequences could be modified, preferably leaving the amino-acid sequence unaltered. According to current regulation, crops that are altered by genome editing, e.g. CRISPR-Cas, are not considered to be GMO (Doudna and Charpentier 2014; Waltz 2016). Therefore, since the modification of a few nucleotides in a sRNA or sRNA target site is sufficient to prevent RNA silencing, targeted modification of immune system components is within our reach. However, during the completion of this review, an important decision was made by the European Court of Justice where they indicate that CRISPR-Cas edited genomes will be classified as GMOs. This decision is a major setback and may slow the progress of crop improvement for a range of traits and will add to the challenges that scientists have to improve crops, including to increase plant resistance.

### How do parasites counteract plant sRNA activity?

Although the RNA silencing mechanism was originally identified as a defence mechanism against viruses, viruses can counteract this defence mechanism by suppressing the host RNA silencing response (Burgyán and Havelda 2011). A range of viral RNA silencing suppressor proteins can impede RNA silencing in the host plant. The mechanistic basis of parasite RNA silencing suppression includes: binding dsRNAs and impeding further processing, preventing silencing signal amplification, interfering with the stabilization of siRNA, and suppression of RISC activity (Alvarado and Scholthof 2009). Likewise, bacteria have also evolved mechanisms to suppress host RNA silencing (Navarro et al. 2008). Furthermore, two secreted proteins from the oomycete *Phytophthora sojae*, the causal agent of root and stem rot of soybean, were also shown to suppress RNA silencing in plants by inhibiting the biogenesis of sRNAs (Qiao et al. 2013; Ye and Ma 2016).

RNA silencing suppressor proteins are translocated from the parasite to the plant (Fig. 2, blue proteins and arrow). Until now, only RNA silencing suppressor proteins from viruses and oomycetes have been identified, but it is likely that other parasites also have them. Still other means to prevent RNA silencing could be anticipated. For example, any RNA molecule with high similarity to a known target site could function as a competing RNA binding site and attenuate sRNA based silencing and interfere with immunity. A parasite might also produce additional (messenger)RNAs that bind sRNAs to sequester and prevent silencing of a target that is required for virulence. Both methods would provide a very specific mode of interference of host gene regulation (relying on sequence similarity to the target site), rather than a more global suppression of multiple sRNAs simultaneously through targeting of the silencing machinery itself. In any case, although the manipulation of sRNA activity by parasites is currently under investigated, it clearly adds another twist to parasite-plant communication.

### Epilogue

In this review we focus on sRNAs that alter plant immunity. Johanna Westerdijk started her position 44 years before the genetic code and messenger RNA were described. What if Johanna could search through the genomes of the fungi in her collection and browse through all the sequenced plant genomes? Even more, what if she could gain insight into the regulation of all the sRNAs and transcripts present in both plant and the fungal genomes. Needless to say, this would allow her to gain insights into the various life styles of fungi on the molecular, genomic and epigenetic level. How different would it have been if she were able to visualize the communication between various organisms on the epigenetic level? Clarifying the “communication sources” e.g. the identification of small RNAs and how they are utilized by both the plant and the parasite to cause resistance or disease can be exploited to develop disease resistant plants in the future.
